# Effectiveness of dispatcher training in increasing bystander chest compression for out‐of‐hospital cardiac arrest patients in Japan

**DOI:** 10.1002/ams2.303

**Published:** 2017-08-07

**Authors:** Taichiro Tsunoyama, Shinji Nakahara, Masafumi Yoshida, Maki Kitamura, Tetsuya Sakamoto

**Affiliations:** ^1^ Department of Emergency Medicine Teikyo University School of Medicine Tokyo Japan

**Keywords:** Bystander, cardiopulmonary resuscitation, emergency medical services, oral guidance by phone, out‐of‐hospital cardiac arrest, training program for emergency call dispatchers

## Abstract

**Aim:**

The Japanese government has developed a standardized training program for emergency call dispatchers to improve their skills in providing oral guidance on chest compression to bystanders who have witnessed out‐of‐hospital cardiac arrests (OHCAs). This study evaluated the effects of such a training program for emergency call dispatchers in Japan.

**Methods:**

The analysis included all consecutive non‐traumatic OHCA patients transported to hospital by eight emergency medical services, where the program was implemented as a pilot project. We compared the provision of oral guidance and the incidence of chest compression applications by bystanders in the 1‐month period before and after the program. Data collection was undertaken from October 2014 to March 2015.

**Results:**

The 532 non‐traumatic OHCA cases were used for analysis: these included 249 cases before and 283 after the guidance intervention. Most patients were over 75 years old and were men. After the program, provision of oral guidance to callers slightly increased from 63% of cases to 69% (*P* = 0.13) and implementation of chest compression on patients by bystanders significantly increased from 40% to 52% (*P* = 0.01). Appropriate chest compression also increased from 34% to 47% (*P* = 0.01). In analysis stratified by the provision of oral guidance, increased chest compressions were observed only under oral guidance.

**Conclusions:**

We found increased provision of oral guidance by dispatchers and increased appropriate chest compressions by bystanders after the training program for dispatchers had been rolled out. Long‐term observation and further data analysis, including patient outcomes, are needed.

## Introduction

Out‐of‐hospital cardiac arrest (OHCA) is a major health concern in industrialized countries. The prognosis among OHCA patients, which is usually quite poor, can be significantly improved by cardiopulmonary resuscitation (CPR) by bystanders before the arrival of emergency medical services (EMS) arrive. Therefore, facilitating chest compressions by bystanders can improve the survival rates among OHCA patients.^1^, ^2^, ^3^ However, only approximately half of witnessed patients receive bystander resuscitation attempts.

Bystanders are reluctant to perform chest compression because of fear of causing harm, fear of litigation, the complexity of performing mouth‐to‐mouth rescue breathing, and the unwillingness to perform mouth‐to‐mouth contact.^4^, ^5^, ^6^ Bystanders’ reluctance may be due to their insufficient knowledge, skills, or confidence. For example, agonal breathing, often present early in cardiac arrests, can be wrongly interpreted as a sign of life by bystanders.^7^

Emergency call dispatchers who receive emergency calls from citizens can directly contact the bystanders and compensate for their lack of skills and confidence by providing oral guidance to facilitate chest compressions by bystanders. Since Carter *et al*.^8^ proposed that a CPR instruction to give five ventilations and chest compressions for 5 min should be given to a bystander by telephone in 1984, many reports have suggested that dispatchers are able to diagnose cardiac arrest over the telephone accurately, and dispatchers’ oral guidance may increase chest compressions by bystanders and improve patients’ outcomes.^9^, ^10^, ^11^, ^12^, ^13^, ^14^, ^15^

To provide appropriate instructions to upset bystanders effectively, dispatchers require a certain level of skills to evaluate the patient's condition, teach resuscitation procedures, and encourage reluctant bystanders using the phone alone. In addition, dispatchers frequently fail to recognize signs of impending cardiac arrests.^9^, ^16^, ^17^, ^18^ Thus, skills training to dispatchers is crucial.

Few studies have evaluated the effectiveness of existing dispatcher training programs. A study in Japan reported that a dispatcher training program in communication with bystanders, along with the prevailing chest‐compression using only CPR as a standard method, increased the incidence of chest compressions from 41% to 56%.^9^

However, the previous studies evaluated training programs in local areas. Since dispatchers’ qualifications and performances differ by region, the Japanese Fire and Disaster Management Agency has developed a standard training material for dispatchers in order to reduce the interregional differences; this has been implemented in model fire departments since 2013.^19^ This study evaluated the effects of the training program in providing oral guidance and in implementation of chest compressions by bystanders.

## Methods

### Study design

This is a retrospective before–after comparison to evaluate the effects of a training program. We compared the provision of telephonic oral guidance by dispatchers to bystanders and the incidence of chest compressions by bystanders in the 1‐month period before and after the training program was implemented, using data obtained from eight model fire departments. The study protocol was approved by the Ethics Committee of Teikyo University School of Medicine (Tokyo, Japan).

### Study setting

Japan has public EMS systems run by municipal governments throughout the country, with a free nationally uniform contact number (119); however, each municipality has its own discretion in managing the system.^20^ Therefore, the EMS systems vary by municipality, with different education levels, qualifications, and working arrangements for the dispatchers as well as the ambulance crews. Ambulance crews and dispatchers include first level emergency medical technicians (EMTs), second level EMTs, and emergency lifesaving technicians (ELSTs). First level EMTs have completed over 135 h of the Basic Recruit Training Course at the Fire Academy. Second level EMTs are individuals who have completed an additional 115 h of the EMT Standard Course following certification as first level EMTs. First and second level EMTs are allowed to perform first aid on patients. Emergency lifesaving technicians who have passed the national board exam after completing the ELST development course are allowed to insert an i.v. line and an adjunct airway, and to use external defibrillators on OHCA patients. After additional training and certification, ELSTs are allowed to carry out tracheal intubation and adrenaline administration.

The Fire and Disaster Management Agency developed a training program for dispatchers to improve the dispatchers’ skills in communication, diagnosis, and instructions. The educational content includes basic medical education, the role of the dispatcher, identification of the urgency and severity (details of signs and symptoms of cardiac arrests, including those of impending arrests), and outline of oral guidance (Doc. S1). The Total Classroom lecture is 7 h 45 min. Simulation training (7 h 45 min) was also included to acquire skills. Eligible participants are EMTs, second level EMTs, and ELSTs. The details of the training program are available on the website.^19^

### Study sites and participants

To evaluate the effectiveness of the program, the training was implemented in 12 model fire departments in 12 municipalities (one department in each municipality). As probability sampling was not feasible, quota sampling based on the population size was used to include various municipalities.

**Figure 1:**
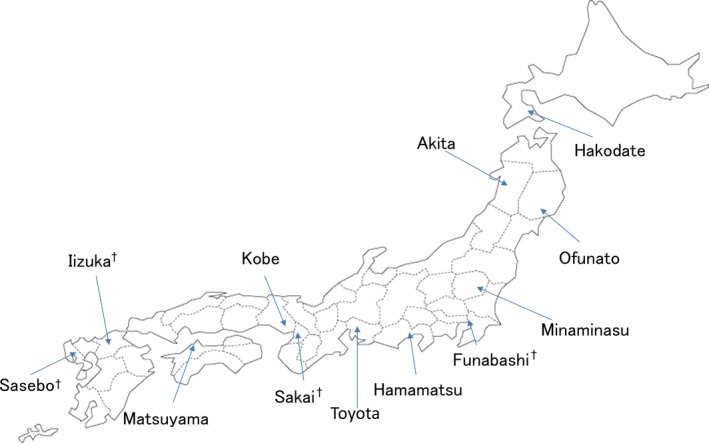
Study sites to evaluate the effectiveness of dispatcher training in increasing bystander chest compression for out‐of‐hospital cardiac arrest patients in Japan, selected by population size. †Excluded from study.

Of the 12 fire departments, we analyzed data from the eight fire departments shown in Table 1 and Figure 1, for which data on provision of oral guidance and incidence of chest compressions by bystanders before and after the training program were available at the time of our analysis. Data was not stored for the other four fire departments.

**Table 1:** Study sites to assess the effectiveness of dispatcher training in increasing bystander chest compression for out‐of‐hospital cardiac arrest patients in Japan, grouped by population size

Population size	Municipalities^a^
≥800,000	Hamamatsu, Sakai,^b^ Kobe
500,000 ≤ to <800,000	Matsuyama, Funabashi^b^
300,000 ≤ to <500,000	Akita, Sasebo,^b^ Toyota
100,000 ≤ to <300,000	Iizuka,^b^ Hakodate
<100,000	Ofunato, Minaminasu

a Each municipality has its own emergency medical services system.

b Four municipalities were excluded from the study.

Our analysis included all consecutive OHCA patients who were transported to the hospital by eight fire departments in the period 1 month before and after the training program. The actual survey period for each fire department was different, but overall, surveys prior to the intervention were carried out from October to December 2014, and surveys following the intervention were carried out from December 2014 to March 2015. Traumatic OHCAs and those who were judged dead at the scene (non‐transported cases) were excluded. We also excluded OHCA cases in which chest compression by the bystander had already started prior to oral guidance, or where oral guidance for CPR was irrelevant because callers and patients were physically separated, and undertaking CPR was impossible (e.g., calls from different locations).

### Data

Ambulance crews collected the on‐scene data using activity logs and a standardized data form. Data collected included the qualifications of the dispatcher (ELST and first and second level EMTs), patient age, sex, who judged cardiopulmonary arrest (the dispatcher by phone or ambulance crews at the scene), provision of oral guidance, chest compressions by the bystanders (including its appropriateness [executed appropriately, executed inappropriately, not executed, or unconfirmed]), and destination (emergency and critical care center, secondary medical care, primary care, or not transported). The appropriateness of chest compressions was evaluated by the ambulance crew at the scene, based on the position, depth (over 5 cm), rate (100/min), and continuation of chest compressions until EMS arrival.

### Statistical analysis

We compared the proportions of implementing dispatcher oral guidance by phone, chest compressions by bystanders, and the appropriate chest compressions between before and after the training. As an effect size, we indicated Cramér's *V*.^21^ Cramér's *V* is typically used to represent the strength of association from χ^2^ analyses (Doc. S2). A *V*‐value of over 0.1 was considered clinically significant. As a statistical test, we used the χ^2^‐test with two‐sided significance level of *P* < 0.05. spss version 16 (SPSS, Chicago, IL, USA) was used in all statistical analyses.

As this was a secondary data analysis, the number of patients was determined in advance. Thus, we calculated power based on the given sample size to indicate the appropriateness of the size, rather than calculating required size. A total of 249 and 283 OHCA patients were included in the analysis before and after the training, respectively. Assuming that appropriate chest compressions implementation rates would be 20% before education, and that this would increase to 30% following education, the power is 0.77 with the type I error of 5%.^22^

## Results

**Figure 2:**
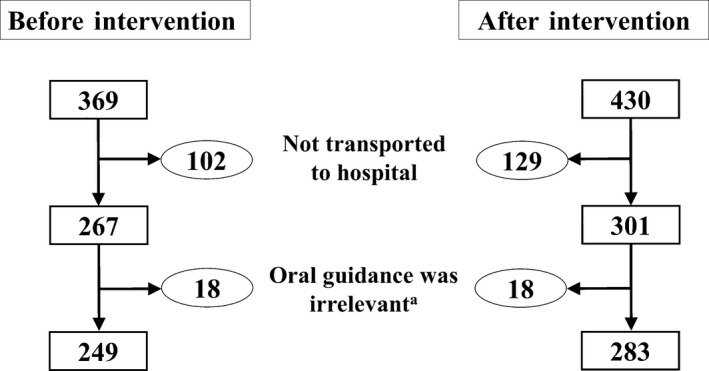
Selection of participants for the study of the effectiveness of dispatcher training in increasing bystander chest compression for out‐of‐hospital cardiac arrest patients in Japan. †Oral guidance for cardiopulmonary resuscitation was irrelevant among these cases because callers and patients were physically separated and intervention was impossible (e.g., calls from different locations).

Of the total of 799 non‐traumatic OHCA cases attended by the eight EMS from October 2014 to March 2015, 568 cases were transported to the hospital. After excluding 36 cases in which CPR instruction was irrelevant, 532 cases remained for analysis, including 249 cases encountered before the training intervention was rolled out, and 283 thereafter (Fig. 2). Table 2 shows the demographic data. Approximately 60% of patients were over 75 years old in both groups. There were slightly more male than female patients in both groups. Approximately 90% of the dispatchers were not ELSTs.

**Table 2:** Patient demographics and prehospital characteristics in an assessment of the effectiveness of dispatcher training in increasing bystander chest compression for out‐of‐hospital cardiac arrest patients in Japan

	Before intervention (*n* = 249) *n* (%)	After intervention (*n* = 283) *n* (%)
Age (years)		
<65	55 (23.7)	54 (19.9)
65–74	30 (12.9)	51 (18.8)
75–84	67 (28.9)	83 (30.5)
85+	80 (34.5)	84 (30.9)
Sex		
Male	127 (51.8)	162 (57.9)
Female	118 (48.2)	118 (42.1)
Qualification of the dispatcher		
ELST	26 (10.4)	35 (12.4)
Second level EMT	133 (53.4)	128 (45.2)
First level EMT	90 (36.1)	120 (42.4)
Destination		
Tertiary level	137 (55.0)	142 (50.2)
Secondary level	112 (45.0)	131 (46.3)
Primary care level	0 (0)	3 (1.1)
Unknown	0 (0)	7 (2.5)

ELST, emergency lifesaving technician; EMT, emergency medical technician.

Table 3 shows a comparison between before and after the training program. The proportions of cases in which cardiac arrest was judged by the dispatchers did not differ. Provision of oral guidance was slightly increased after the intervention (63% versus 69%). Cases of implementation of chest compressions by bystanders and appropriate chest compressions significantly increased after the intervention (*V* = 0.12 for both).

**Table 3:** Comparison between before and after the implementation of a training program to improve the skills of emergency call dispatchers in providing oral guidance on chest compression to bystanders who have witnessed out‐of‐hospital cardiac arrests

	Before intervention (*n* = 249)	After intervention (*n* = 283)	*V*‐value^b^	*P*‐value^a^
	*n* (%)	*n* (%)		
Recognition of cardiopulmonary arrest				
By the dispatcher	203 (81.5)	235 (83.0)	0.02	0.648
By the ambulance crews	46 (18.5)	48 (17.0)		
Provision of oral guidance				
Yes	157 (63.1)	195 (68.9)	0.09	0.132
No	72 (28.9)	76 (26.9)		
Chest compression already started	20 (8.0)	12 (4.2)		
Chest compressions at EMS arrival				
Executed	77 (40.1)	133 (52.2)	0.12	0.011
Unexecuted	115 (59.9)	122 (47.8)		
Unconfirmed	57	28		
Appropriate chest compressions				
Yes	66 (34.4)	119 (46.7)	0.12	0.009
No/inappropriate	126 (65.6)	136 (53.3)		
Unconfirmed	57	28		

a χ^2^‐Test with two‐sided significance level of *P* < 0.05.

b Cramér's *V* (*V*‐value >0.1 clinically significant).

EMS, emergency medical services.

Table 4 shows the status of implementation of chest compressions as stratified according to whether CPR was carried out with or without oral guidance. After the intervention, cases of chest compressions and appropriate chest compressions increased only under oral guidance (*V* = 0.11 for both cases). Although cases where chest compressions had already started before oral guidance was given, and cases involving appropriate chest compressions before oral guidance were increased, the number of these cases were small.

**Table 4:** Subgroup analyses stratified by the provision of oral guidance on chest compression by emergency call dispatchers to bystanders who have witnessed out‐of‐hospital cardiac arrests

	Before intervention *n*/*N* a (%)	After intervention *n*/*N* a (%)	*V*‐value^b^
With oral guidance			
Chest compressions (+)	65/120 (54.2)	112/172 (65.1)	0.11
Appropriate chest compressions (+)	56/120 (46.7)	99/172 (57.6)	0.11
Unconfirmed	37	23	
Without oral guidance			
Chest compressions (+)	9/67 (13.4)	11/72 (15.3)	0.03
Appropriate chest compressions (+)	7/67 (10.4)	10/72 (13.9)	0.05
Unconfirmed	5	4	
Chest compression already started			
Chest compressions (+)	3/5 (60.0)	10/11 (90.9)	0.37
Appropriate chest compressions (+)	3/5 (60.0)	10/11 (90.9)	0.37
Unconfirmed	15	1	

a Denominators indicate the total number of the respective subgroup, excluding those with unconfirmed information on chest compression.

b Cramér's *V* (*V*‐value >0.1 clinically significant).

## Discussion

We evaluated the effects of a dispatcher training program on chest compression implementation by bystanders in eight municipalities. After the training program, oral guidance by dispatchers to bystanders increased, and the incidence of chest compressions by bystanders, as well as the cases with appropriate chest compressions, increased even though each magnitude of effect was small. The incidence of chest compressions and appropriate chest compressions mainly increased under oral guidance by dispatchers.

Previous studies have reported the effectiveness of a comprehensive dispatcher training program on the incidence of chest compressions by bystanders and on patient outcomes.^9^, ^23^ A study in Ishikawa, Japan, evaluated a project that introduced a new protocol and training for dispatchers on how to provide oral guidance to bystanders to perform chest compression, and how to recognize cardiac arrests with information obtained over the telephone, e.g., agonal breathing, emesis, and convulsions.^9^ After the training intervention, the dispatchers’ provision of instructions to bystanders and chest compressions given by bystanders significantly increased (from 42% to 62%, and from 41% to 56%, respectively); the 1‐year neurologically intact survival also increased (from 1.9% to 2.8%). Another study in the same area reported that the training project improved the ability of the dispatchers to identify cardiac arrest accurately using telephonically obtained information: the sensitivity increased (50.3% versus 72.9%), but the specificity did not change (99.6% versus 99.8%).^23^

Our findings are consistent with these previous findings. In addition, the implementation rates of chest compression were higher than the rates of 31–55% reported in other parts of country.^24^, ^25^ A standardized protocol and training can increase the use of telephonically provided oral guidance by dispatchers, and the provision of chest compression by bystanders increased only among those who received such guidance. As no increase was observed among those who did not receive the guidance, it is unlikely that the increased bystander‐administered chest compressions only indicate a temporal trend that is not related to training. Although the previous findings were derived from relatively small areas, our findings were derived from various municipalities throughout the country, from small townships to urban areas, which may imply the advantages of implementing a standardized nationwide protocol and training program.

Education for dispatchers is thought to improve the knowledge, practical ability, and motivation of dispatchers. However, there are problems of securing the time and instructors for education. As various innovations are needed to solve the problem, it is necessary to formulate a policy to introduce education according to the size of the fire department.

This study was subject to several limitations. First, because of the lack of information on patient outcomes in this study, we could not show the actual data leading to improved survival rate due to increased chest compressions by citizens. However, as the association between dispatcher‐assisted CPR and favorable outcomes is well known, the increased incidence of chest compressions by bystanders under the oral guidance provided by dispatchers is a reliable indicator for evaluating the effectiveness of dispatcher training. Second, this study evaluated short‐term effects of the program in increasing chest compressions by bystanders. Further long‐term observational studies are required as training effects may wane in the long term; these should ideally determine how long training effects are retained, and how often refresher training is necessary. Finally, the study sites were not selected using a random sampling procedure. However, we included various types of municipalities using quota sampling; thus, various types of dispatchers were trained. Our findings may be applicable to other areas of Japan that are somewhat similar to several of our study sites, even though our samples did not exactly represent the whole country.

## Conclusion

In this study, we have reported increased provision of oral guidance by dispatchers to callers, chest compressions by bystanders to OHCA patients, and appropriate chest compressions by bystanders after the roll‐out of a dispatcher training program. Long‐term observation and further data analysis, including analysis of patient outcomes, are required.

## Disclosure

The study protocol was approved by the Ethics Committee of Teikyo University School of Medicine. There is no applicable number for the registry or registration of the study.

Conflict of interest: None declared.

## Supporting information


**Doc. S1.** Content of the standardized training curriculum for emergency call dispatchers developed by the Fire and Disaster Management Agency, Japan.
**Doc. S2.** Cramér's *V* is computed by taking the square root of the χ^2^ statistic divided by the sample size and the minimum dimension minus 1.
