# Supplementary material for: Effectiveness of dispatcher training in increasing bystander chest compression for out‐of‐hospital cardiac arrest patients in Japan
Source: Acute Med Surg. 2017 Aug 7;4(4):439–45. doi: 10.1002/ams2.303 (PMC5649305; doi:10.1002/ams2.303)
Supplement: Supplementary file 1 — Doc. S1. Content of the standardized training curriculum for emergency call dispatchers developed by the Fire and Disaster Management Agency, Japan. Doc. S2. Cramér's V is computed by taking the square root of the χ2 statistic divided by the sample size and the minimum dimension minus 1. [file AMS2-4-439-s001.docx]

Appendix A

| Curriculum | Contents |
| --- | --- |
| General aspects of EMS system | - Review of statistics: trend of EMS utilization in the past, present, and future - Emergency medical care system: critical care medical centres, secondary care centres, and other hospitals - Activities of EMS at the scene - Supervision by physicians |
| General dispatcher skills | - Dispatchers’ role in EMS - Communications with callers - Communications with ambulance teams - Management of urgent situation: communication and collaboration with pumpers and hospitals |
| Medical knowledge | - Anatomy and physiology - Pathological condition leading to cardiopulmonary arrest - Cardiopulmonary resuscitation procedures - AED utilization - Airway management |
| Identification of urgent symptoms via the telephone | - How to identify agonal breathing and seizure as a sign of cardiac arrest on the phone - How to identify shock - How to identify dyspnoea - How to identify unconsciousness |
| Protocols for oral guidelines for each condition | - Cardiopulmonary resuscitation - Removal of airway foreign bodies - Control bleeding - Burn - Finger(s) Amputation |
| Skill training | Simulation training using scenarios |

Appendix B

Cramér's V is computed by taking the square root of the chi-squared statistic divided by the sample size and the minimum dimension minus 1:

Cramer’s V is


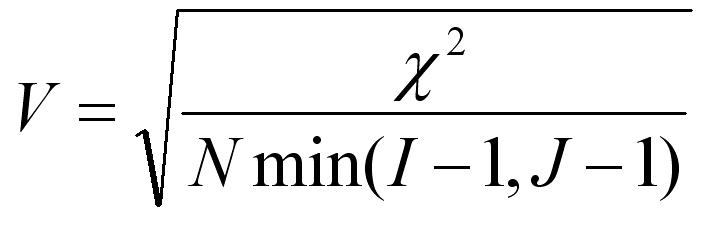
, where I and J are the numbers of rows and columns, and N is the total number of events.

| Magnitude of Effect Size | Cramer’s V |
| --- | --- |
| Small | 0.1 |
| Medium | 0.3 |
| Large | 0.5 |
