# Supplementary material for: Effectiveness of dispatcher training in increasing bystander chest compression for out‐of‐hospital cardiac arrest patients in Japan
Source: Acute Med Surg. 2017 Aug 7;4(4):439–45. doi: 10.1002/ams2.303 (PMC5649305; doi:10.1002/ams2.303)
Supplement: Supplementary file 2 [file AMS2-4-439-s002.docx]

**Supporting Information**

Appendix S1. Contents of the standardized training curriculum for emergency call dispatchers developed by the Fire and Disaster Management Agency, Japan

Appendix S2

Cramér's V is computed by taking the square root of the chi-squared statistic divided by the sample size and the minimum dimension minus 1:

Cramer’s V is


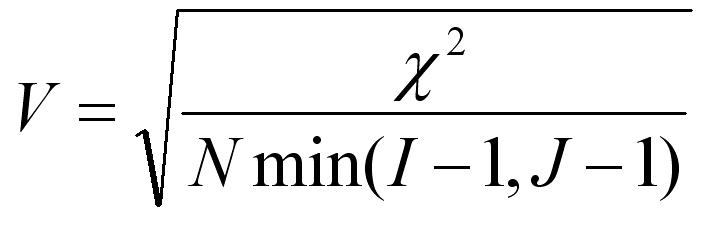
, where I and J are the numbers of rows and columns, and N is the total number of events.
